# Supplementary material for: Preparation and Evaluation of Topically Applied Azithromycin Based on Sodium Hyaluronate in Treatment of Conjunctivitis
Source: Pharmaceutics. 2019 Apr 15;11(4):183. doi: 10.3390/pharmaceutics11040183 (PMC6523157; doi:10.3390/pharmaceutics11040183)
Supplement: Supplementary file 1 [file pharmaceutics-11-00183-s001.pdf]

pharmaceutics

Supplementary Materials: Preparation and Evaluation of Topically Applied Azithromycin Based on Sodium Hyaluronate in Treatment of Conjunctivitis

Qian Chen, Chun Yin Jiang Ma, Jiasheng Tu and Yan Shen

1. Materials and Method

1.1. Degradation Kinetics of AZI

A stock solution containing 1% AZI was used to investigate the degradation kinetics of AZI. The stock solution (1 mL) was separately diluted to 10 mL using phosphate buffer solutions with different pH values (1, 3, 5, 7, 10) and incubated at 75 °C for 2 h, 4 h, 6 h, 12 h and 24 h. The incubate solutions (0.5 mL) was then diluted ten times with the mobile phase to obtain test solutions. The actual pH values of each test solution were monitored. Moreover, the concentration of AZI (C_{AZI}) at each time points was quantified by HPLC-UV. Then, $\ln C_{AZI}$ was plotted against time (t) to analyze the apparent hydrolysis rate constant (K) and the kinetic order of degradation reactions under different pH. Subsequently, the pH where AZI was the most stable (pH_m) was obtained from the lnk-pH curve, which was located at the lowest point of this curve.

1.2. Cytotoxicity Assay

Human corneal epithelial (HCE-2) cells were cultured in Dulbecco's modified Eagle's medium (DMEM) which contained 10% (*v*/*v*) fetal calf serum, 100 IU/mL penicillin G and streptomycin, and 2 mM L-glutamine at 37 °C with 5% CO_2 and air humidified atmosphere. HCE-2 cells were exposed to different concentrations of BZK solution, AZI-SH eye drops and commercial AZI eye drops diluted by medium for 24 h. Each concentration was tested in triplicates. DMSO (0.5%) served as the solvent control. The cell viability was then detected by MTT assay. MTT solution (20 μL, 5 mg/mL) was added into each well and incubated for 4 h. Then, the MTT-containing medium was removed and 200 μL of DMSO was added. The absorbance was measured at 490 nm by microplate reader (MD Spectramac M3, USA). The cell inhibition rate was calculated using the following Equation (1).

$$\text{Cell inhibition rate (\%)} = \left(1 - \frac{I_{sample} - I_{blank}}{I_{control} - I_{blank}}\right) \times 100\% \quad (1)$$

where I_{sample} and $I_{control}$ are the mean absorbance values of tested group and control group, respectively. I_{blank} is the absorbance value of the medium.

2. Results and Discussion

The cytotoxicity of AZI-SH eye drops toward human corneal epithelial cells in vitro was investigated via MTT assay. The results of MTT assay showed that AZI-SH eye drops had minimal toxicity in the range of 0.05–50 μg/mL, while BZK caused severe cytotoxicity. As illustrated in Figure S1, the cytotoxicity of these three groups increased significantly along with the increase in BZK concentration. The increase in toxicity was due to the addition of BZK. The IC_{50} of AZI-SH eye drops was 29.09 μg/mL, which was 1.35 times and 3.85 times higher than that of commercial AZI eye drops (21.60 μg/mL) and BZK solution (7.56 μg/mL), respectively.

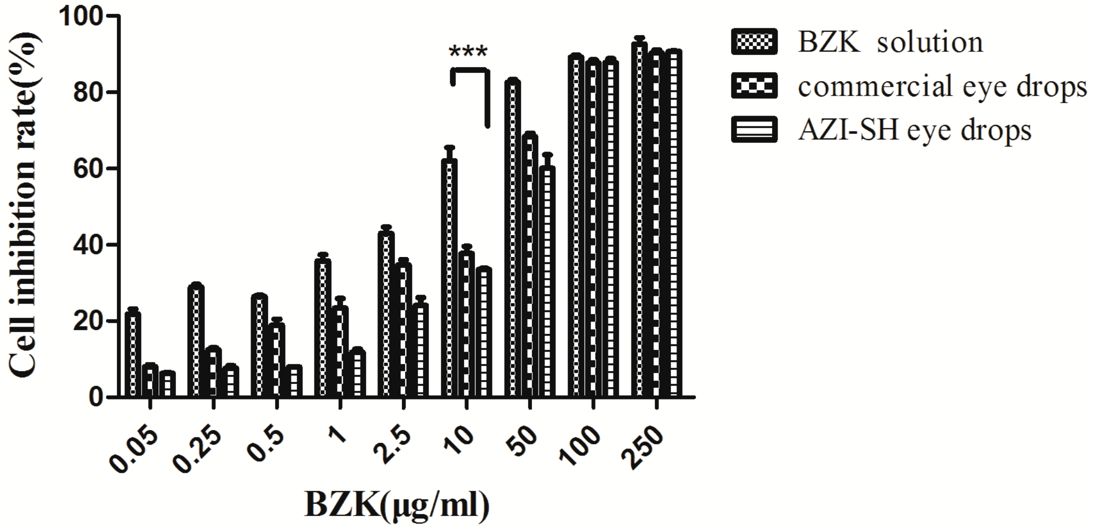

Figure S1. Cell viability against HCE-2 cells determined by MTT assay, (***$P < 0.01$).

Table S1. Scores for grading the severity of ocular lesions/grading system in the ocular irritation tests.

**Cornea**	
lesion	score
No opaque	0
Scattered or diffuse area - details of iris clearly visible	1
Easily discernible translucent areas, details of iris slightly obscured	2
Opalescent areas, no details of iris visible, size of pupil barely discernible	3
Opaque, iris invisible	4
**Iris**	
lesion	Score
Normal	0
Folds above normal, congestion, swelling, circumcorneal injection (any one or all of these or combination of any thereof), pupil still reacting to light (sluggish reaction is positive)	1
No reaction to light, hemorrhage; gross destruction (anyone/all of these)	2
**Conjunctiva**	
Lesion	Score
A. Redness (refers to palpebral conjunctiva only)	
Normal	0
Vessels definitely injected above normal	1
More diffuse, deeper crimson red, individual vessels not easily discernible	2
Diffuse beefy red	3
B. Chemosis	
No swelling	0
Any swelling above normal (includes nictitating membrane)	1
Obvious swelling with partial eversion of the lids	2
Swelling with lids about half closed	3
Swelling with lids about half closed to completely closed	4
C. Discharge	
No discharge	0
Any amount different from normal (does not include small amount observed in inner canthus of normal animals)	1

Discharge with moistening of the lids and hairs just adjacent to the lids	2
Discharge with moistening of the lids and considerable area around the eye	3
Total maximum	16

Table S2. Evaluation criteria for eye irritation reaction.

Scores	Evaluation
0–3	Nonirritant
4–8	Slightly irritant
9–12	Moderate irritant
13–16	Severe irritant

Table S3. Influence of 3% azithromycin eye drops on rabbit papillary diameter (cm, *n* = 4).

Group	Animal No.	1	2	3	4
AZI	After administration (0min)	0.6	0.5	0.5	0.5
	After administration (25min)	0.5	0.6	0.6	0.5
	After administration (8h)	0.6	0.5	0.7	0.5
	After administration (24h)	0.7	0.5	0.6	0.6
	After administration (48h)	0.6	0.6	0.6	0.5
Saline	After administration (0min)	0.6	0.6	0.5	0.5
	After administration (25min)	0.7	0.5	0.6	0.5
	After administration (8h)	0.5	0.6	0.7	0.6
	After administration (24h)	0.6	0.7	0.5	0.5
	After administration (48h)	0.6	0.6	0.6	0.7
